# Analysis of Stress-Responsive Gene Expression in Cultivated and Weedy Rice Differing in Cold Stress Tolerance

**DOI:** 10.1371/journal.pone.0132100

**Published:** 2015-07-31

**Authors:** Caroline Borges Bevilacqua, Supratim Basu, Andy Pereira, Te-Ming Tseng, Paulo Dejalma Zimmer, Nilda Roma Burgos

**Affiliations:** 1 Universidade Federal de Pelotas, Pelotas, Capão do Leão, Rio Grande do Sul, Brazil; 2 Department of Crop, Soil, and Environmental Sciences, University of Arkansas, Fayetteville, Arkansas, United States of America; Louisiana State University Agricultural Center, UNITED STATES

## Abstract

Rice (*Oryza sativa* L.) cultivars show impairment of growth in response to environmental stresses such as cold at the early seedling stage. Locally adapted weedy rice is able to survive under adverse environmental conditions, and can emerge in fields from greater soil depth. Cold-tolerant weedy rice can be a good genetic source for developing cold-tolerant, weed-competitive rice cultivars. An in-depth analysis is presented here of diverse *indica* and *japonica* rice genotypes, mostly weedy rice, for cold stress response to provide an understanding of different stress adaptive mechanisms towards improvement of the rice crop performance in the field. We have tested a collection of weedy rice genotypes to: 1) classify the subspecies (ssp.) grouping (*japonica* or *indica*) of 21 accessions; 2) evaluate their sensitivity to cold stress; and 3) analyze the expression of stress-responsive genes under cold stress and a combination of cold and depth stress. Seeds were germinated at 25°C at 1.5- and 10-cm sowing depth for 10d. Seedlings were then exposed to cold stress at 10°C for 6, 24 and 96h, and the expression of cold-, anoxia-, and submergence-inducible genes was analyzed. Control plants were seeded at 1.5cm depth and kept at 25°C. The analysis revealed that cold stress signaling in *indica* genotypes is more complex than that of *japonica* as it operates via both the CBF-dependent and CBF-independent pathways, implicated through induction of transcription factors including *OsNAC2*, *OsMYB46* and *OsF-BOX28*. When plants were exposed to cold + sowing depth stress, a complex signaling network was induced that involved cross talk between stresses mediated by CBF-dependent and CBF-independent pathways to circumvent the detrimental effects of stresses. The experiments revealed the importance of the CBF regulon for tolerance to both stresses in *japonica* and *indica* ssp. The mechanisms for cold tolerance differed among weedy *indica* genotypes and also between weedy *indica* and cultivated *japonica* ssp. as indicated by the up/downregulation of various stress-responsive pathways identified from gene expression analysis. The cold-stress response is described in relation to the stress signaling pathways, showing complex adaptive mechanisms in different genotypes.

## Introduction

About two-thirds of the global land area undergo freezing cycles, or is in permafrost, and 42% of agricultural land can experience freezing temperatures up to -20°C [1]. To survive under these conditions, plants have developed specialized mechanisms involving adaptive morphological, physiological, and biochemical changes. Low temperature stress impairs crop growth and development, causing crop yield losses [2]. Plants exhibit differential responses to chilling (0–15°C) or freezing (< 0°C) temperatures. Plants in temperate regions acclimate to cold temperatures [3] through various biochemical and physiological changes [4, 5], enabled by increased expression of cold-responsive genes that eventually leads to alteration in lipid composition of membranes and accumulation of osmolytes [6]. In contrast, tropical and sub-tropical plants lack cold-adaptation mechanisms and are highly sensitive to cold stress.

Survival under cold stress is a highly complex process involving various metabolic pathways and cellular compartments [7]. Conventional breeding methods have produced some cold-tolerant cultivars, but progress is slow owing to the complexity of stress tolerance traits, low genetic variance of yield components under stress conditions, and absence of suitable selection criteria. Locally adapted cold-tolerant cultivars are therefore needed. Exploring genomic resources to develop cold-tolerant crops is necessary.

Rice (*Oryza sativa* L.) feeds more than half of the global population [8], second only to wheat (http://www.idrc.ca/EN/Resources/Publications/Pages/ArticleDetails.aspx?PublicationID=565). Rice is grown in a wide range of environments (tropical, sub-tropical, temperate) but it is a tropical C3 crop [9], which yields best under warm temperatures and high solar radiation while still remaining inefficient [10]. Therefore, in non-tropical environments (and in high altitudes), low temperature stress during crop establishment and reproductive stage causes significant yield losses. Cold stress around planting time impairs rice emergence and hampers early seedling growth and development [11]. This makes rice vulnerable to competition from weeds, especially weedy rice. A competitive variety is a strong component of an integrated weed management program [12–13], and work on varietal improvement for competitive ability needs to be pursued.

When cold temperature occurs at the reproductive stage, it causes spikelet sterility [14–15] and reduces spikelet number and overall panicle volume [16]. In the rice-producing region of northern Japan, rice yield can decline by up to 60% in a cold year [17]. In the USA, cold stress is a major constraint to the establishment and growth of early-planted rice and is detrimental to yield of late-planted rice. There is good potential for varietal improvement in cold tolerance because of the observed genetic diversity in rice germplasm with respect to this trait [18]. About 40 quantitative trait loci (QTLs), in various combinations, impart cold tolerance in various rice varieties [19], with the *japonica* ssp. generally more cold-tolerant than *indica* ssp. [20–21].

Exposure of plants to cold temperatures disrupts the expression of its full genetic potential owing to direct inhibition of cellular metabolism, or indirectly via cold-induced oxidative and other abiotic stresses. Acclimation to cold is achieved by chronic exposure to cold temperatures [22] through accumulation of cold-adaptive genes with time. One such gene is *FRO1* (*FROSTBITE 1*). The *Arabidopsis fro1* (*frostbite1*) mutant exhibits susceptibility to chilling and freezing stress through impaired expression of cold responsive (COR) genes and a substantial accumulation of reactive oxygen species (ROS) [23]. Thus, cold stress signaling in plants is significantly affected by ROS accumulation and ABA, which are secondary cellular messengers. However, some weedy rice ecotypes may have a better cold adaptation mechanism because weedy rice seedlings grow faster than cultivated rice even during early planting in temperate climate when cold stress often occurs (Burgos, NR pers. observation). This warrants investigation of cold tolerance or cold adaptation mechanisms in weedy rice.

Difference in cold tolerance is a result of centuries of adaptation in cold, rice-growing regions [24]. Cold tolerance is a complex trait, involving many genes, and is highly genotype-dependent. For example, the transcription factor *MYBS3* mediates cold stress signaling and cold tolerance in rice via a *MYBS3*-dependent pathway that allows long-term cold adaptation, and acts in conjunction with the short-term cold stress signaling pathway mediated by *DREB1/CBF* [25]. The rice *FATTY ACID DESATURASE 2* (*OsFAD2*) also enhances cold tolerance by maintaining membrane fluidity during low temperature stress [26]. Other cold-tolerance mechanisms have also been reported.

Outside of Asia, Brazil and the USA are among the highest rice-producing countries (http://globalriceproduction.com/). Although the USA primarily grows *japonica* cultivars, yield depression by cold stress still occurs periodically. The attempt to avoid cold stress narrows the rice planting window and creates constraints on farming resources. Brazil, on the other hand, grows primarily *indica* rice, which is generally cold-sensitive; and farmers thus have to contend with problems such as reduced or variable rice emergence and reduced yield due to cold stress [27–30]. Rice growers in regions like these need to plant rice early to synchronize the onset of reproductive stage with the highest solar radiation period and obtain the highest possible yield. Weedy rice may grow faster than a cold-sensitive cultivar at early planting, making the weed even more adaptive and competitive than the crop. Thus, improving cold tolerance in rice seedlings could boost rice production tremendously. Cold-tolerant *japonica* rice lines [31] are excellent sources of cold-tolerance genes that could be accumulated in desirable high yielding cultivars; sourcing these traits from *japonica* lines to improve cold tolerance in *indica* varieties has been challenging because of high sterility among *indica* x *japonica* hybrids. An alternative is to source cold tolerance traits for *indica* cultivars from different *indica* lines. The majority (>90%) of weedy rice in the USA are *indica* [32–33] and some *indica* weedy ecotypes may be cold-tolerant.

Seeding depth exacerbates the effect of cold stress on rice establishment, as it affects seedling vigor. Rice cultivars, having been selected for uniform and quick emergence, do not emerge from deep in the soil profile. The weedy ecotypes, however, exhibit a wide diversity in ability to emerge from a greater depth [34–35].]. The US weedy ecotypes that could emerge from deep placement are most likely *indica* because of the dominance of weedy *indica* ssp. in the region; such ecotypes have high seedling vigor and may be able to withstand cold stress at the seedling stage [34]. Therefore, screening of rice germplasm for seedling cold tolerance is an important first step in finding resources for cold tolerance improvement. The present study investigates diverse weedy rice genotypes and rice cultivars for tolerance to cold stress, and makes a comparative analysis of the response mechanisms between weedy and cultivated rice in the *indica* and *japonica* subspecies. To dissect the cold tolerance mechanisms, we conducted real time quantitative-PCR (qPCR) analysis of gene expression in response to cold stress and a combination of cold and depth stress compared to normal temperature, of *indica* and *japonica* genotypes differing in cold sensitivity. Data from this experiment will be a resource in improving cold stress tolerance in rice and its competitive ability with weeds in early planting in temperate and sub-tropical regions.

## Methods

### Plant Materials and Subspecies Identification

Weedy rice seed samples, comprised of 21 weedy red rice accessions and rice cultivars for subspecies (ssp.) identification, were collected from rice fields in Arkansas, the largest rice-growing state in the southern USA, and from Rio Grande do Sul, Brazil. Weedy rice seed collection was done primarily in collaboration with Extension Agents with permission from growers and, occasionally, with the growers themselves. Some rice cultivars were obtained from rice breeders, but the majority were requested from the US Germplasm Resources Information Network-National Plant Germplasm System (GRIN-NPGS) (http://www.ars-grin.gov/npgs/aboutgrin.html). No endangered species were involved nor impacted by this activity. The phenol test [32] was conducted to determine the ssp. genotype (*japonica* or *indica*) of the materials to be used in subsequent experiments. The phenol test is based on the color change of rice hulls or endosperm upon exposure to phenol as a result of polyphenol oxidase (PPO) activity, where *japonica* varieties show no color change (a negative response), while *indica* varieties and wild *Oryza* species take on a dark brown or black coloration [32]. Intact grains soaked in 1.5% non-buffered aqueous phenol for 48h were compared with nontreated seeds from the same seed lot. A positive reaction to phenol is indicated by color change of the grain, from light to dark (S1 Fig). The *indica* genotype has positive reaction to phenol. The experiment was conducted with one positive control (1602) and one negative control, ‘Spring’. Samples showing negative phenol reaction were tested twice for verification.

### Plant Materials and Growth Conditions for Stress Tolerance Phenotyping

A subset of samples used in the phenol test (31) was phenotyped for cold- and seeding-depth stress tolerance. To overcome dormancy, the seeds were soaked in 1.5% NaOCl for 24h. Seeds were rinsed with deionized water, surface sterilized with 0.1% HgCl_2_ for 20min then planted at different depths in pots, filled with vermiculite.

For cold stress only, the seeds were planted 1.5cm deep and placed in a growth chamber in the dark for 14d. Control plants were germinated at 25°C; cold-stressed plants were grown at alternating temperatures of 18°C/13°C, 16/8h cycle, in the dark. For depth stress only, the seeds were planted at 1.5-, 5-, 10-, and 15-cm depth and germinated at 25°C in the dark for 14d. For the combined stress treatments (depth + cold), seeds were planted at different depths (5, 10, and 15cm); the cold-stressed plants were germinated at 18°C/13°C, 16/8-h cycle, in the dark, while the controls were kept at 25°C. At 14d, the shoot lengths of five seedlings per growth condition per accession were measured. The experiments were repeated. Shoot length reduction (%) was then calculated relative to the control plants of each accession and used as a measure of sensitivity to cold and seeding depth stress [36–38]. Accessions showing <50% reduction in shoot length under cold or depth stress, relative to their corresponding non-stressed plants, were deemed cold- or depth-tolerant. The cold-tolerant and selected sensitive accessions were used in the gene expression analysis experiment. Phenotyping was done at 18°C/13°C to allow plants to germinate from deep seeding and identify a broader spectrum of cold stress-tolerant genotypes. We learned in preliminary experiments that at 10°C, few accessions could germinate at normal seeding depth and none could germinate when planted deeper.

For gene expression analysis in response to cold stress only, pregerminated seeds were planted at 1.5-cm depth and allowed to grow for 10d at 25°C. Thereafter, cold stress was initiated by transferring plants to 10°C [39–40] while the control plants were kept at 25°C. Leaves of stressed and non-stressed plants were harvested at 6, 24, and 96h of incubation in cold temperature. Each treatment had three replicates. The same procedure was followed for the cold + seeding-depth stress experiment, except that the pregerminated seeds were planted at a depth of 10cm, grown at 25°C for 10d, then incubated at 10°C for 6, 24, and 96hr before harvesting leaf tissues. Control plants were kept at 25°C. Cold stress-induced gene expression was studied at 10°C to capture genes most strongly involved in adaptation to chilling stress.

### Gene Expression Analysis

Total RNA was isolated using Trizol reagent (Invitrogen) from stress-tolerant and-sensitive genotypes identified from the phenotyping experiment. cDNA synthesis was conducted using 2μg total DNAse-treated RNA using GoScript Reverse Transcription System (Promega). The qRT-PCR experiments were conducted using GoTaq qPCR Master Mix (Promega), gene-specific primers (Table 1 and S1 Table), and ubiquitin as standard with three biological replicates and each replicate divided into two technical replicates in a CFX-96 Bio-Rad thermocycler (Bio-Rad). Increasing temperature (0.5°C 10 s^-1^) from 55°C to 95°C was used for melt curve analysis. Un-transcribed RNA was also run as negative control. The relative difference in expression for each sample in individual experiments was determined by normalizing the Ct value for each gene against the Ct value of ubiquitin and was calculated relative to the respective control samples as calibrator using the equation 2-ΔΔCt [41]. The data from qPCR analysis was imported into TM4 microarray software suite, normalized using GC-RMA algorithm to generate the heat map. The average of three biological replicates was used to obtain each expression value [42].

**Table 1:** Summary of genes analyzed.

Gene	Trigger	Result	Reference
***OsNAC2***	Salt, Drought, Cold	Abiotic Stress Tolerance	[80]
***OsDREB2C***	Salt, Mannitol and Cold	Thermotolerance in *Arabidopsis thaliana* (*AtDREB2C*)	[93–94]
***OSMYB46***	Drought	Overexpression led to ectopic deposition of secondary cell wall in nonsclerenchymatous cells	[95–96]
***OsFBOX28***	Cold	None	[97]
***OVP1***	Cold	Improves Cold tolerance in rice	[69]
***Rab 16A***	Salt, Drought, Cold	Improves Salt tolerance in rice	[42,98]
***GLP***	ABA, Cold, H_2_O_2_	None	[99]
***GDH***	Salt, Cold, Drought	None	[64,100]
***APX1***	Salt, Cold	Overexpression in rice showed enhanced tolerance to cold stress at booting stage	[101–102]
***ADH1***	Anoxia, Cold	*ADH1* overexpression in *Arabidopsis* had no effect on flooding tolerance but was essential for survival under anoxic conditions	[89,92,103]
***EXPA 7***	Anoxia, Submergence	None	[88,104]
***EXPA 12***	Anoxia, Submergence	None	[89]
***RAMY3D***	Anoxia, Cold	None	[90]
***ERF 70***	Anoxia, Salt, Drought	Overexpression in *Arabidopsis thaliana* gives tolerance to osmotic stress	[89,104,105]
***ERF 68***	Anoxia, Drought, Salt	None	[89,106,107]
***SUB1b***	ABA, Drought, Submergence	None	[89,108]

## Results and Discussion

### Classification of Weedy and Cultivated Rice Subspecies

Weedy red rice has diverse phenotypic characteristics, possessing traits that are common to both wild and cultivated rice. A diverse set of rice genetic resources was used here to examine the diversity of tolerance to abiotic stress, and the molecular basis of such traits, in this weedy relative of rice. Previous research classified the US weedy rice strains as either wild- or crop-like, but the adaptive phenotypic variation of weedy rice has not been fully characterized. Rice is classified into two major subspecies *indica* and *japonica*, which differ in a suite of classical diagnostic traits, i) seedling reaction to KClO_3_, ii) seedling survival in cold temperatures, iii) grain apiculus hair length, and iv) phenol reaction. The phenol test used to distinguish the rice cultivars and weedy accessions separated the 21 tested genotypes into 6 *japonica* and 15 *indica* subspecies (Table 2). The four rice cultivars were all *japonica*. Of the 15 Arkansas weedy rice accessions, 13 (87%) were *indica*. Previous studies also have shown that *japonica* weedy rice is rare in the USA. Analysis of weedy rice accessions from the southern USA ricebelt (Arkansas, Mississippi, Louisiana, Texas) using simple sequence length polymorphic (SSLP) markers, showed that 19 of 21 accessions (90%) belonged to the *indica* ssp. group [33]. More recently, the phenol reaction test on 108 weedy rice accessions from southern USA showed that 96% were *indica* [32]. Thus, there are abundant *indica* genotypes that are potential sources of desirable traits for crop improvement, especially of *indica* cultivars.

**Table 2:** Weedy and cultivated rice (*Oryza sativa* L.) genotypes subspecies grouping by phenol test following the method of Gross et al., 2009.

Accession code	Hull color	Origin	Rice type	Subspecies	GPS Coordinates
**ARK-5-SH**	Strawhull	Arkansas, AR, USA	weedy red rice	*Indica*	91°22.246' W, 34°07.220' N
**CHI08-C-SH**	Strawhull	Chicot, AR, USA	weedy red rice	*Indica*	91°03.680' W 35°25.958' N
**CON-1-BH**	Blackhull	Conway, AR, USA	weedy red rice	*Indica*	92°11.326' W, 34°71.388' N
**CRA08-D-SH**	Strawhull	Craighead, AR, USA	weedy red rice	*Indica*	90°58.976' W, 35°45.791' N
**DES-1-SH**	Strawhull	Desha, AR, USA	weedy red rice	*Indica*	91°27.695' W, 33°46.151' N
**GRE08-D-SH**	Strawhull	Greene, AR, USA	weedy red rice	*Indica*	90°46.677' W, 36°01.764' N
**LEE08-C-SH**	Strawhull	Lee, AR, USA	weedy red rice	*Indica*	90°02.237' W, 34°45.791' N
**LIN08-B-BH**	Blackhull	Lincoln, AR, USA	weedy red rice	*Indica*	91°42.018' W, 33°54.839' N
**LIN08-B-SH**	Strawhull	Lincoln, AR, USA	weedy red rice	*Indica*	91°42.008' W, 33°54.854' N
**LIN08-C-SH**	Strawhull	Lincoln, AR, USA	weedy red rice	*Indica*	91°42.529' W, 33°57.653' N
**LON-3-SH**	Strawhull	Lonoke, AR, USA	weedy red rice	*Indica*	91°52.475' W, 34°52.258' N
**1200**	Strawhull	Pelotas, RS-Brazil	weedy red rice	*Indica*	52°26.483' W, 31°49.024' S
**1602**	Strawhull	Pelotas, RS-Brazil	weedy red rice	*Indica*	52°25.761' W, 31°48.758' S
**RAN-2-SH**	Strawhull	Randolph, AR, USA	weedy red rice	*Indica*	90°56.703' W, 36°12.510' N
**LAW08-B-SH**	Strawhull	Lawrence, AR, USA	weedy red rice	*Indica*	90°54.981' W, 35°58.380' N
**PRA08-D-BH**	Blackhull	Prairie, AR, USA	weedy red rice	*Japonica*	91°26.955' W, 34°44.770' N
**WOO-5-BH**	Strawhull	Woodruff, AR, USA	weedy red rice	*Japonica*	91°17.156' W, 35°00.315' N
**3011(Diamante)**	Strawhull	Chile	rice cultivar	*Japonica*	Not available
**Oro**	Strawhull	Chile	rice cultivar	*Japonica*	Not available
**Hayayuki**	Strawhull	Japan	rice cultivar	*Japonica*	Not available
**Spring**	Strawhull	USA	rice cultivar	*Japonica*	91°25.134' W, 37°28.567' N

### Phenotyping for Cold Tolerance

The rice plants growing in cold temperatures show various symptoms of stress-induced damage such as reduced leaf expansion, chlorosis, and impaired growth. Reduction in seedling growth is a good indicator for cold tolerance during seed germination and seedling emergence. Tolerance to cold stress at this period is invaluable in achieving faster crop establishment and better competitive ability with weeds. The three rice *japonica* cultivars (Hayayuki, Spring, and 3011) had mean shoot lengths of 105, 98, and 41mm, respectively, when planted at normal depth (1.5cm) under normal temperature (25°C) for 14d (Table 3). ‘Hayayuki’ from Japan [43], has been used as cold-tolerant reference in other experiments and so was included here together with the more recently commercialized cold-tolerant, tropical *japonica* ‘Spring’ from Arkansas and another putative cold-tolerant *japonica* cultivar from Chile ‘3011’. The 10 weedy rice accessions tested had shoot lengths ranging from 55 to 191mm, with PRA08-D-BH as the fastest growing ecotype under normal temperature. When subjected to cold stress, at the normal 1.5-cm seeding depth, shoot length was reduced 30% to 95% relative to the non-stressed controls (Table 3). The cultivars and weedy rice accessions showing <50% reduction in shoot length under cold stress were classified as cold-tolerant. With this criterion, only two *indica* weedy rice (GRE08-D-SH and 1602) and one *japonica* cultivar (Spring) were deemed tolerant to cold stress at the seedling stage. The cold-tolerant 3011 (from Chile) turned out to be sensitive to the level of cold stress implemented (18°C/13°C day/night) at this growth stage. It should be noted that rice can be tolerant to cold stress at later growth stages, but sensitive at the seedling stage. It should also be noted that the tolerant genotypes were not totally immune to cold, showing 29% to 37% shoot length reduction relative to the non-stressed plants, with Spring (reference cold-tolerant genotype) showing 37% shoot growth reduction. The most cold-sensitive ones, such as PRA08-D-BH, had 95% reduction in shoot length. This indicates that the cold tolerance trait is not common among *indica* genotypes, which makes the discovery of tolerant *indica* genotypes of greater importance for the development of cold-tolerant *indica* cultivars.

**Table 3:** Seedling growth of weedy and cultivated rice (*Oryza sativa* L.) genotypes in response to cold stress under normal seeding depth of 1.5cm.

Accession code	Origin	Rice type	Subspecies	Shoot length at 25°C (mm)^a^		Shoot length reduction under cold stress (%)^b^	Cold tolerance^c^
**CHI08-C-SH**	Chicot, AR, USA	weedy red rice	*Indica*	122	b	94	sensitive
**CRA08-D-SH**	Craighead, AR, USA	weedy red rice	*Indica*	162	a	95	sensitive
**GRE08-D-SH**	Greene, AR, USA	weedy red rice	*Indica*	103	b	29	tolerant
**LEE08-C-SH**	Lee, AR, USA	weedy red rice	*Indica*	173	a	92	sensitive
**LIN08-B-BH**	Lincoln, AR, USA	weedy red rice	*Indica*	55	c	92	sensitive
**LIN08-B-SH**	Lincoln, AR, USA	weedy red rice	*Indica*	94	b	91	sensitive
**LIN08-C-SH**	Lincoln, AR, USA	weedy red rice	*Indica*	138	a	95	sensitive
**1200**	Pelotas, RS-Brazil	weedy red rice	*Indica*	186	a	95	sensitive
**1602**	Pelotas, RS-Brazil	weedy red rice	*Indica*	76	b	31	tolerant
**PRA08-D-BH**	Prairie, AR, USA	weedy red rice	*Japonica*	191	a	95	sensitive
**3011(Diamante)**	Chile	rice cultivar	*Japonica*	41	c	82	sensitive
**Hayayuki**	Japan	rice cultivar	*Japonica*	105	b	30	tolerant
**Spring**	USA	rice cultivar	*Japonica*	98	b	37	tolerant

^a^ Means followed by the same letter differ by Scoot-Knott test at 5% level, using square root data transformation in the form: y + 0.5-sqrt (y + 0.5). Shoot length was the average of five seedlings measured per replication per genotype, at 14d of incubation.

^b^ Seeds were germinated in the dark at 18/13°C, 16/8h cycle temperature fluctuation. Shoot length reduction was calculated relative to control seedlings grown at 25°C; reduction = [(control—cold stress)/control]*100.

^c^Accessions showing <50% reduction in shoot length under cold stress were deemed cold-tolerant. Means were compared using t-test at *P*≤0.05

### Tolerance to Seeding Depth

Planting depth is a key factor that influences seedling vigor, and eventually crop yield [44]. Rapid and uniform emergence of seedlings provides crops with temporal and spatial advantages to compete with weeds, and is therefore necessary for maximizing yield [45]. It has been shown that sowing depth influences the ability of soybean to emerge from the soil and establish a uniform stand [46]. Weedy rice has the versatility to emerge from greater depth, over a prolonged period than the crop, and thus becomes a big problem. Under normal temperature, the weedy rice accessions and cultivars generally were not affected by seeding at 5-cm depth (S2 Fig). However, 60% (6 of 10) of the accessions tested showed at least 50% reduction in shoot length when planted at 10-cm depth. In general, shoot growth declined further when seeds were planted at 15-cm depth. Altogether, 7 of 10 genotypes tested were classified tolerant to deep sowing; of these, the most depth-tolerant were the weedy rice accessions LEE08-C, CRA08-D, and LIN08-B3. However, when planted deep (10cm) and subjected to cold stress (18°C/13°C, 16/8-h temperature fluctuation cycle, dark-incubated), all accessions lost any tolerance to deep sowing (S3 Fig).

### Genetic Mechanisms for Cold Stress Acclimation in Cultivated and Weedy rice

Low temperature causes cell dehydration, resulting in damage to the plasma membrane, which is the most detrimental effect of chilling stress [47]. Low temperature induces secondary stress messengers like abscisic acid and ROS that in turn induce calcium signatures, effecting cold stress signaling in plants. The existence of some cold-tolerant ecotypes here and elsewhere [48], suggests some diversity in stress adaptation mechanism among weedy genotypes, which might be useful to incorporate in rice cultivars.

The exposure of plants to harsh environmental conditions triggers a wide variety of genes that are responsible for modulation of stress-induced signal transduction cascade. The stress-induced genes are classified into two groups [49–51]: i) those that encode proteins that endow stress tolerance including antifreeze proteins, detoxification enzymes, heat-shock proteins or osmotins, among others; and ii) transcription factors that regulate the expression of other genes and induce tolerance. A genetic-based approach uses natural variation among rice cultivars and weedy rice, and the analysis of differentially expressed genes in response to stress. The greatest challenge in post-genomic research is to determine the function of candidate genes that are identified by analysis of their expression profiles upon exposure to cold stress.

**Fig 1:**
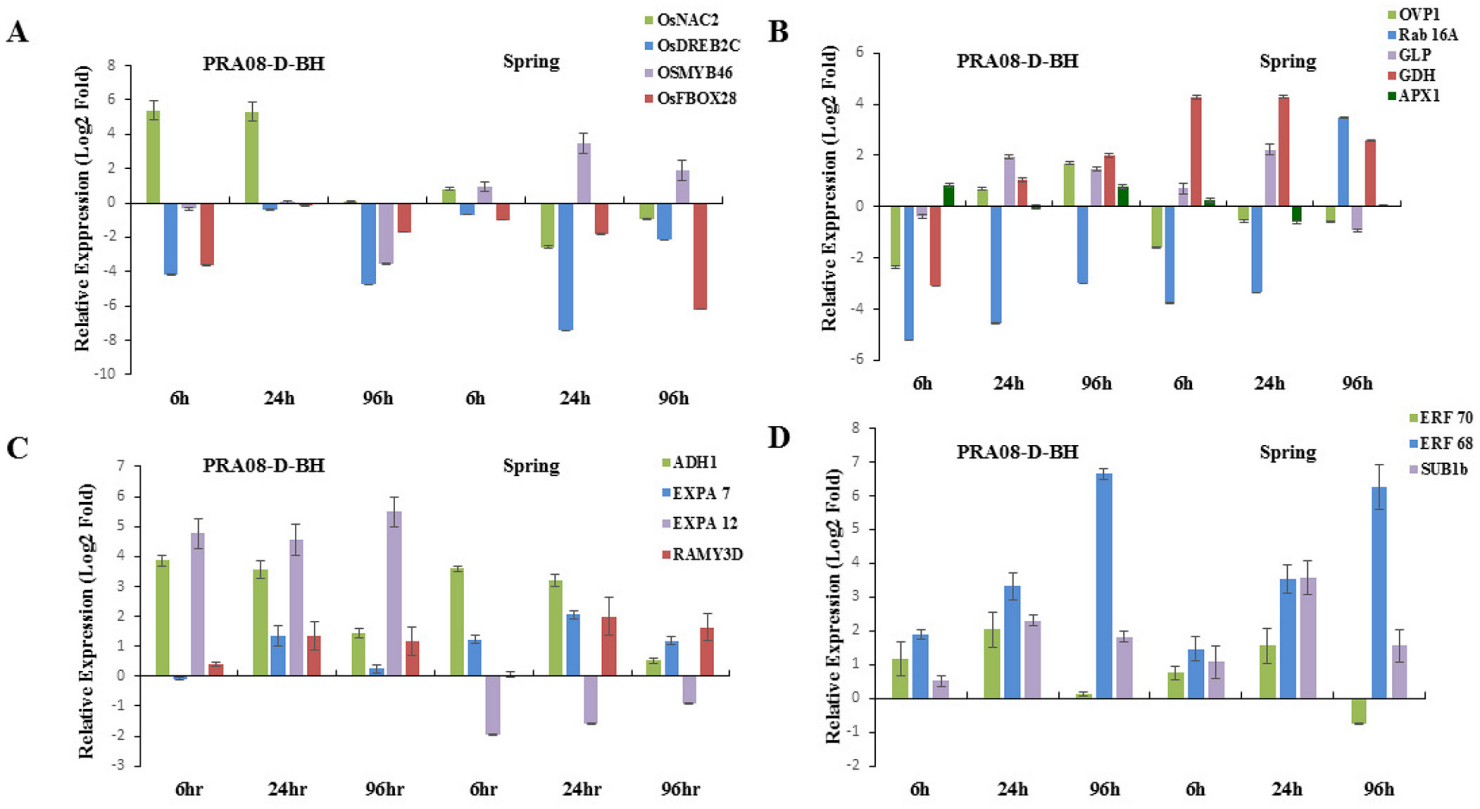
Relative gene expression under cold stress (10°C) in 10-d-old seedlings of sensitive (PRA08-D-BH) and tolerant (Spring) *japonica* rice. The data are means of three replicates ± SE. A) Response of transcription factors: *OsNAC2*, *OsDREB2C*, *OsMYB46* and *OsF-BOX28*. B) Transcript accumulation of defense- and metabolism-related genes: *OVP1*, *Rab16A*, *APX1*, *GLP*, *GDH*. C) Genes related to cell expansion and carbohydrate metabolism: *ADH1*, *EXPA7*, *EXPA12 and RAMY3D*. D) Pattern of expression of ERFs: *ERF70*, *ERF68* and *SUB1B*.

**Fig 2:**
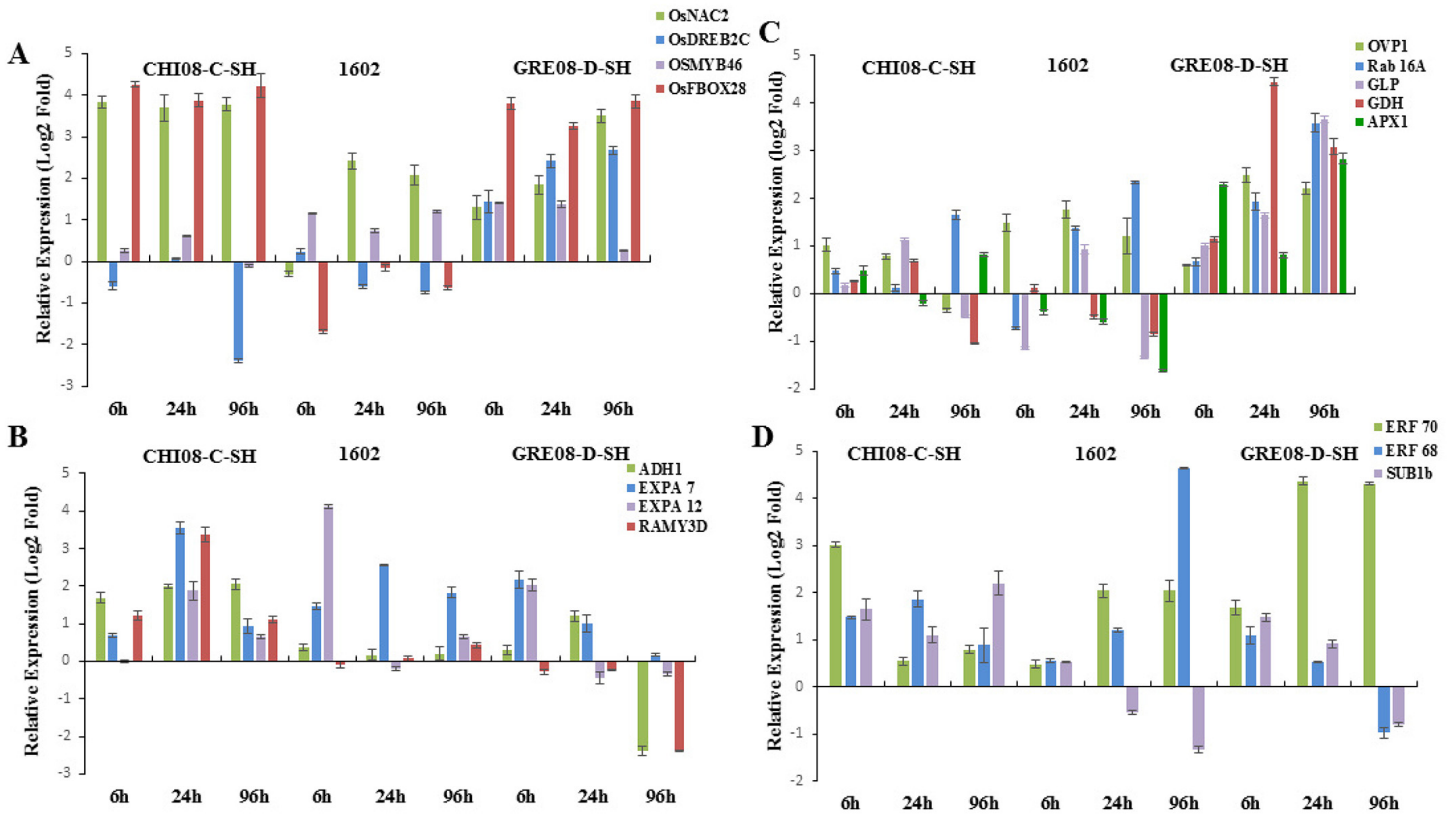
Relative expression of genes under cold stress (10°C) in 10-d-old seedlings of sensitive (CHI08-C-SH) and tolerant (1602 and GRE08-D-SH) *indica* rice. The data are means of three replicates ± SE. A. Response of transcription factors: *OsNAC2*, *OsDREB2C*, *OsMYB46* and *OsF-BOX28*. B. Transcript accumulation of defense- and metabolism-related genes: *OVP1*, *RAB16A*, *APX1*, *GLP*, *GDH*. C. Genes related to cell expansion and carbohydrate metabolism: *ADH1*, *EXPA7*, *EXPA12 and RAMY3D*. D. Expression patterns of ERFs: *ERF70*, *ERF68* and *SUB1B*.

When temperature approaches freezing, osmotically active water typically moves out of the cells, and the osmotic potential of the remaining unfrozen intracellular and intercellular fluid increases. Severe chilling stress causes multiple forms of membrane damage as a consequence of freeze-induced cellular dehydration including expansion-induced lysis and cross talk between stresses [47]. To gain insight into the stress tolerance mechanism in *indica* and *japonica* rice genotypes we studied the expression of key stress-regulated genes *RAB16A* (Lea group of proteins), *H* ^*+*^ *PYROPHOSPHATASE (OVP1)*, *GERMIN*-like proteins (*GLP*s), *GLUTAMATE DEHYDROGENASE* (*GDH*) and *ASCORBATE PEROXIDASE* (*APX1*) (Figs 1B and 2B). *GLPs* have been reported previously to be activated by abiotic stresses like salinity and high temperature [52–54]. *GLP*s are stress-responsive genes that are known to encode proteins with SOD activity, thereby helping in scavenging superoxide radicals [53–54]. In barley, six subfamilies of *GLP*s have been well characterized and are found to encode proteins with oxalate oxidase or superoxide dismutase activity [53–54]. Moreover, GLPs have also been reported to build a defensive barrier during barley emergence [55]. Among the plant materials studied, the tolerant *japonica* cultivar Spring showed induction of *GLP*s at 6h, induced maximally at 24h and reduced completely at 96h, while the sensitive *japonica* weedy genotype from the USA [PRA08-D-BH] showed induction only at 24h and maintained its level at 96h (Fig 1B). The cold-sensitive and tolerant weedy *indica* CHI08-C-SH and GRE08-D-SH showed induction at 6h and maximizing at 24h and 96h, respectively. Another tolerant weedy *indica* rice (1602) showed induction only at 24h (Fig 2B).

The immediate response of plants to stress is accumulation of ROS, which creates ionic imbalance within the cell. To characterize the involvement of ROS scavenging enzymes we studied the expression of *APX1*. The expression of this cytosolic enzyme is governed by H_2_O_2_ accumulation, ABA level, and leaf water status. *APX1* is highly upregulated in roots and shoots in response to salinity, osmotic, and heat stress [56]. Ascorbate peroxidases are involved in stress tolerance, being central components of the hydrogen peroxide scavenging networks in plants. *Arabidopsis* plants deficient in APX1 are highly susceptible to oxidative stress [57–58], suggesting that APX1 is linked to ROS signaling or scavenging. Our research showed that the cold-sensitive *japonica* (PRA08-D-BH) has early induction of *APX1* (Fig 1B) which declined at 24h and increased again at 96h. On the other hand, the tolerant *japonica* (Spring) had a barely perceptible induction of *APX1* at 6h of cold stress, and disappeared at 24h, suggesting the presence of a strong defense mechanism. The sensitive *indica* (CHI08-C-SH) showed the same expression pattern of *APX1* under cold stress as the sensitive *japonica* PRA08-D-BH (Fig 2B). The tolerant *indica* genotypes from Brazil and the USA showed different expression patterns of *APX1*. The tolerant *indica* weedy rice from USA, GRE08-D-SH, showed high induction of *APX1* at 6h, with no detectable expression at 24h and induced maximally again at 96h. On the contrary, the tolerant *indica* weedy rice from Brazil (1602) had no detectable expression. The maximal induction of *APX1* at 96h suggests that cold stress also induces secondary stress responses such as anoxia and cold-induced dehydration due to ROS generation. The role of APX1 in cold stress tolerance differed between *japonica* and *indica* and between *indica* genotypes from Brazil and the USA suggesting the presence of different ROS scavenging machinery involving APX1 and other antioxidant enzymes that reflects localized adaptations.

The role of phytohormone ABA-responsive gene expression in response to cold stress was studied through the expression of the *RAB16A* gene, a well-characterized member of the *LEA* gene family [59]. *RAB16A* is induced by abiotic stresses such as salinity and ABA treatment [60–61], which cause dehydration, and plays a crucial role in stabilizing the cell membrane and preventing denaturation of proteins; thus, endowing stress tolerance [62]. RAB16A also binds to iron and acts as antioxidant under abiotic stress [61]. The tolerant *indica* genotype; GRE08-D-SH showed early induction of *RAB16A* (6h) with maximum transcript accumulation at 96h of cold incubation while another tolerant genotype, 1602, showed induction at 24h and increased maximally at 96h (Fig 2B). The sensitive *indica* genotype had lower level of *RAB16A* transcript than the tolerant ones, which was downregulated at 96h. The expression of *RAB16A* was not induced in the tolerant *japonica* genotype until 96h of incubation and had no detectable expression, at any time, in cold-sensitive *japonica* (Fig 1B). These results suggest the activity of an ABA-dependent tolerance pathway in the *indica* genotypes, which might not be functional in the *japonica* ssp.

Glutamate dehydrogenase (GDH) (EC 1.4.1.2) is a key enzyme involved in amino acid biosynthesis catalyzing the reductive amination of 2-oxoglutarate (2OG) and the oxidative deamination of Glu *in vitro* under nitrogen-limiting conditions and is upregulated under abiotic stresses [63–64]. ROS plays a critical role in the signaling pathway for *GDH* expression and protease activation that also contributes to intracellular hyper-ammonia. GDH also has a crucial role in germinating seeds and senescing leaves, converting amino acids into transport compounds with a low C/N ratio [65]. The expression of *GDH* is induced upon exposure to salinity stress; it is likely involved in ammonia detoxification and protease inhibition by activating protein synthesis [66]. The *GDH* gene was strongly induced at 6h of cold stress in the cold-tolerant *japonica* cultivar (Spring) with a slight decrease at 96h, while the sensitive weedy *japonica* (PRA08-D-BH) showed induction only at 24h. The sensitive weedy *indica* (CHI08-C-SH) showed lower transcript accumulation of *GDH* at 6 and 24h compared with the tolerant weedy *indica* (GRE08-D-SH), which maintained higher transcript accumulation and reaching maximum at 24h. However, the cold-tolerant weedy *indica* rice (1602) from Brazil had barely detectable transcript accumulation at 6h, which then subsided at 24h. Our observation supports a stress-protective role of GDH along with its physiological role and the aminating activity of the anionic isozymes of GDH. Our data also indicate that the involvement of GDH in cold stress adaptation differs between genotypes and between regions.

Plants have two distinct proton pump systems in the vacuolar membrane H^+^-ATPase (V-ATPase, EC 3.6.1.3) and H^+^- translocating inorganic pyrophosphatase (V-PPase EC 3.6.1.1) [67]. The proton pump creates an electrochemical potential gradient across the membrane, thereby helping in translocation of solutes across the membrane. Earlier it has been shown that overexpression of H^+^-*TRANSLOCATING INORGANIC PYROPHOSPHATASE* (*OVP1*) in rice imparts tolerance to cold stress [67–69]. Here, we observed that the induction of *OVP1* expression under cold stress occurred only in *indica* weedy rice, with stronger expression in tolerant genotypes (Fig 2B). The maximum expression was observed at 24h with slight reduction at 96h in GRE08-D-SH; in 1602, the maximum induction was observed at 24h and was completely downregulated at 96h. The sensitive weedy *japonica* (PRA08-D-BH) showed late induction at 24h with no detectable transcript accumulation in the tolerant *japonica* cultivar (Spring). These observations suggest a role for *OVP1* in maintaining the integrity of the proton pump machinery and oxidative phosphorylation. Our observations suggest the presence of a different cold tolerance mechanism in *japonica* than in *indica* rice.

### Role of Transcription Factors in Stress Tolerance

Cold stress-activated ROS accumulation induces several transcription factors (TFs) that operate via the ABA-dependent and ABA-independent pathways, eliciting cold tolerance. Cold stress induces the accumulation of CBFs (C-repeat binding factors, also known as DEHYDRATION-RESPONSIVE ELEMENT-BINDING protein or DREBs), which bind to the *cis*-elements in the promoters of cold responsive genes and activate their expression [68,70], consequently inducing the expression of genes involved in ROS detoxification, membrane transport, osmolyte biosynthesis, phosphoinositide metabolism [49,71–72]. The DREB2C protein binds to the C-repeat/dehydration response element (CRE) in vitro and possesses transcriptional activity. It has also been shown that *Arabidopsis* plants overexpressing *DREB2C* are cold- or thermo- tolerant [73]. The expression of *DREB2C* gene in tolerant *indica* US weedy rice was induced early and at high levels in a time-dependent manner, but no detectable expression was observed in the other tolerant *indica* rice (1602) nor in the sensitive weedy *indica* wherein a low transcript accumulation was observed only at 6h and then disappeared (Fig 2A). *DREB2C* also was not detected in cold-stressed *japonica* genotypes (Fig 1A). Thus, our data support previous research, which showed that *DREB2C* overexpression endowed tolerance to freezing and heat stress [74] with only transient induction by other abiotic stresses [75].

Another important class of TFs that play a role in cold-induced gene expression are members of the MYB family. Previous reports have shown that *hos10-1* (R2R3-type MYB) mutant *Arabidopsis* plants are susceptible to cold stress, even though they have induced expression of *CBF*s. Moreover, abiotic stresses like cold, salinity and drought induce *OsMYB3R-2* (an R1R2R3 MYB) that positively regulates stress tolerance via a CBF-independent pathway [76]. A characteristic feature of cold stress tolerance is the maintenance of cell wall integrity, as cells lose water owing to freezing-induced dehydration. Previously it has been shown that plants reduce the expression of cell wall-modifying enzymes like XTHs (Xyloglucan Endotransglucosylase/Hydrolases) in *Vigna radiata*, deduced from reduced hypocotyl elongation [77]. *OsMYB46* is a unique TF directly regulating the biosynthetic genes for major components of the secondary wall, as well as TFs in the biosynthesis pathway [78]. Both the tolerant weedy *indica* genotypes showed early and high *OsMYB46* transcript accumulation up to 24h with a slight reduction observed at 96h in GRE08-D-SH in comparison to the sensitive one (Fig 2A). The tolerant *japonica* cultivar showed early induction of *OsMYB46* and sustained expression for 96h of cold stress, while no induction was observed in the sensitive weedy *japonica* genotype (Fig 1A). The elevated level of *OsMYB46* upon stress imposition suggests that the tolerant genotypes attempt to alleviate the stress and minimize stress impact by fortifying the cell wall.

NAC transcription factors are an important group of transcriptional regulators in plants whose members play diverse roles in stress tolerance [79]. In previous reports *SNAC1*, *SNAC2*, *OsNAC45* genes were shown to be induced by salinity, drought, and cold stress, and overexpression of these conferred stress tolerance [80–82]. In this study, although the tolerant *indica* weedy rice (GRE08-D-SH) showed a progressive increase in *OsNAC2* transcript accumulation and reaching maximum at 96h (at 24h for 1602), the transcripts generally were at lower levels than the sensitive *indica* weedy rice (CHI08-C-SH) (Fig 2A). The sensitive weedy *japonica* (PRA08-D-BH) also maintained a high transcript level up to 24h of cold treatment. In contrast, the tolerant *japonica* cultivar (Spring) showed low accumulation of *OsNAC2* transcripts at 6h that disappeared thereafter (Fig 1A).

Overexpression of F-box proteins confers cold tolerance to plants. The tolerant and sensitive USA *indica* weedy rice maintained a high *OsFBOX28* transcript level over the stress period (Fig 2A), while no detectable transcript accumulation was observed in the *japonica* subspecies or the tolerant *indica* weedy rice from Brazil (Fig 1A). This suggests that genes like *OsNAC2* and *OsFBOX28* are responsive to cold stress in some genotypes, but such response is not always observed in a cold-tolerant phenotype.

Overall, we observed that tolerance to cold stress in *indica* rice occurs via the ABA-dependent as well as the ABA-independent pathway (CBF-mediated) while cold tolerance in *japonica* occurs primarily via the ABA-dependent pathway. The cold stress tolerance mechanisms are different between the weedy rice ecotypes from Brazil and USA origins and between *japonica* and *indica* genotypes.

### Cross-talk Between Stresses

Cold stress induces accumulation of reactive oxygen species represented by peroxide, peroxyl radicals, and others. Due to the lack of efficient ROS scavenging machinery in the cell, plants continue accumulating the negatively charged radicals that consequently leads to oxygen deficiency, giving rise to an anoxic condition. Expansins are proteins that are responsible for shoot elongation under anoxic conditions [83–84]. Previously it has been shown that the rice *EXPANSIN* genes *EXPA7* and *EXPA12* are transiently upregulated under anoxia, presumably facilitating shoot elongation [85]. The *japonica* ssp. genotypes (PRA08-D-BH, Spring) showed high transcript accumulation of *EXPA7*, which declined with time (Fig 1C). Transcript accumulation of *EXPA12* was higher earlier in the sensitive than the tolerant *japonica* and declined with time. The *indica* ssp. genotypes (CHI08-C-SH, sensitive; 1602, tolerant) showed high transcript accumulation of *EXPA7* at 24h and declining at 96h of cold stress, whereas GRE08-D-SH (tolerant) showed high transcript accumulation at 6h then declining thereafter (Fig 2C). Thus, *EXPA12* was highly induced early and downregulated at 24h or later in tolerant weedy *indica* rice, while upregulation was delayed (at 24h) in the sensitive weedy *indica* rice. These data show that transient upregulation of *EXPANSINS* also occur in cold stress (as in anoxia) and that the cold-tolerant genotypes have a different mechanism of controlling the expression of cell wall-modifying proteins than the sensitive ones.

Anoxia induces the level of several *AP2/ERF* genes in *Arabidopsis* [85]. Anoxic condition induced by submergence also triggers the expression of *SUB1A*, *SUB1B*, and *SUB1C*, respectively belonging to the B-2 subgroup of the ERF proteins [86]. To elucidate the role of ERFs in cold stress-induced anoxia we analyzed the expression of *ERF70*, *ERF68* and *SUB1B*. *ERF70* was generally highly induced in all genotypes while *ERF68* showed the opposite pattern of expression. (Figs 1D and 2D). In the sensitive *indica* genotype *ERF70* and *ERF68* were induced maximally at 6h and 24h, respectively. In the tolerant genotype (1602) both TFs were induced with time while the tolerant GRE08-D-SH, showed maximum induction of *ERF70* and *ERF68* at 24h and 6h, respectively. *SUB1B* was induced early in the sensitive and tolerant *japonica* ecotypes then declined with time (Fig 1D). The same pattern was observed in both tolerant *indica* weedy rice, but induction was sustained with time in the sensitive *indica* (Fig 2D). This group of genes does not seem to contribute to cold tolerance in *japonica* cultivar, but may contribute to cold tolerance in *indica* weedy rice.

### Role of Alpha Amylase and Alcohol Dehydrogenase

Alpha amylase (RAMY3D) is a key enzyme involved in the hydrolysis of starch into metabolizable sugars, providing energy for shoot elongation [87] and is known to be a gene induced by starvation [85,88]. *RAMY3D* has been reported to be induced by cold stress as well as anoxia in hyacinth and rice shoots [89–90]. Rapid degradation of starch in the anoxic rice shoots suggests a significant role for this gene in starch metabolism [89]. *RAMY3D* was induced early at 6h of cold stress in both sensitive and tolerant *japonica* and approached basal level at 96h (Fig 1C). *RAMY3D* was induced at all time-points in the sensitive *indica*, but generally was down-regulated in tolerant *indica* genotypes (Fig 2C). This gene does not contribute to cold tolerance in the *japonica* cultivar, but suppression of amylase activity may contribute to cold tolerance in *indica* weedy rice by conserving energy. ADH is a key enzyme triggered under low oxygen conditions, involved in redirecting the switch from aerobic to anaerobic fermentation [91]. Previous reports have shown that *ADH* is highly induced by cold stress and anoxia in maize and rice seedlings [89, 92]. The increased expression of *ADH* probably helps in the regeneration of NAD+ by reducing acetaldehyde to ethanol thereby providing an alternative pathway to glycolysis and in turn preventing cellular acidosis. We observed that *ADH* expression follows a similar pattern as that of *RAMY3D*. This suggests that cold stress induces an anoxic condition where plants respond by increasing sugar metabolism and switching to anaerobic respiration.

### Combined Effects of Cold and Deep Sowing Stress on Stress-Responsive Gene Expression

**Fig 3:**
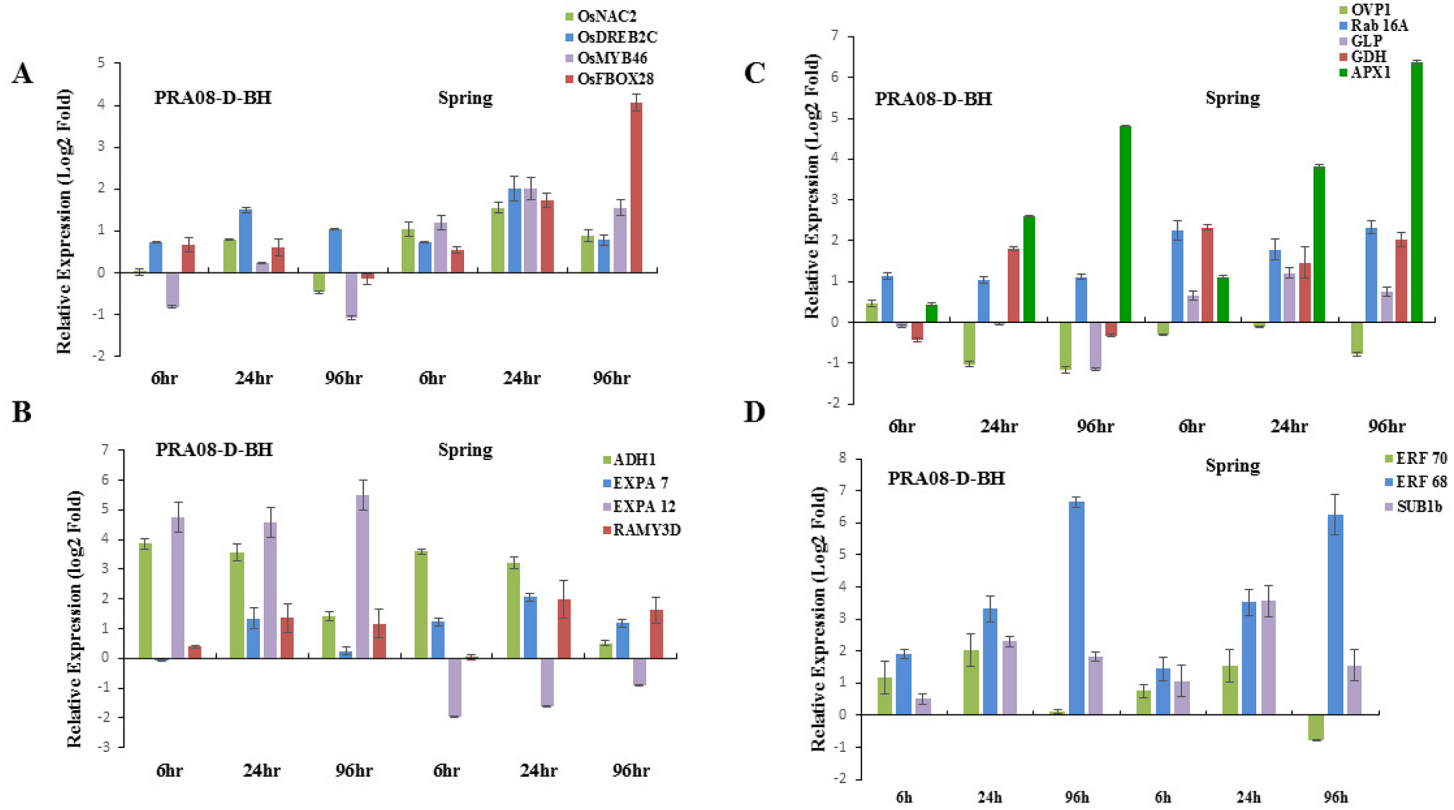
Relative expression of genes in response to combined cold stress (10°C) and seeding depth stress (10cm) in 10-d-old seedlings of sensitive (PRA08-D-BH) and tolerant (Spring) *japonica* rice. The data are means of three replicates ± SE. A. Response of transcription factors: *OsNAC2*, *OsDREB2C*, *OsMYB46* and *OsF-BOX28*. B. Transcript accumulation of defense- and metabolism-related genes: *OVP1*, *RAB16A*, *APX1*, *GLP*, *GDH*. C. Genes related to cell expansion and carbohydrate metabolism: *ADH1*, *EXPA7*, *EXPA12 and RAMY3D*. D. Expression patterns of ERFs: *ERF70*, *ERF68* and *SUB1B*.

**Fig 4:**
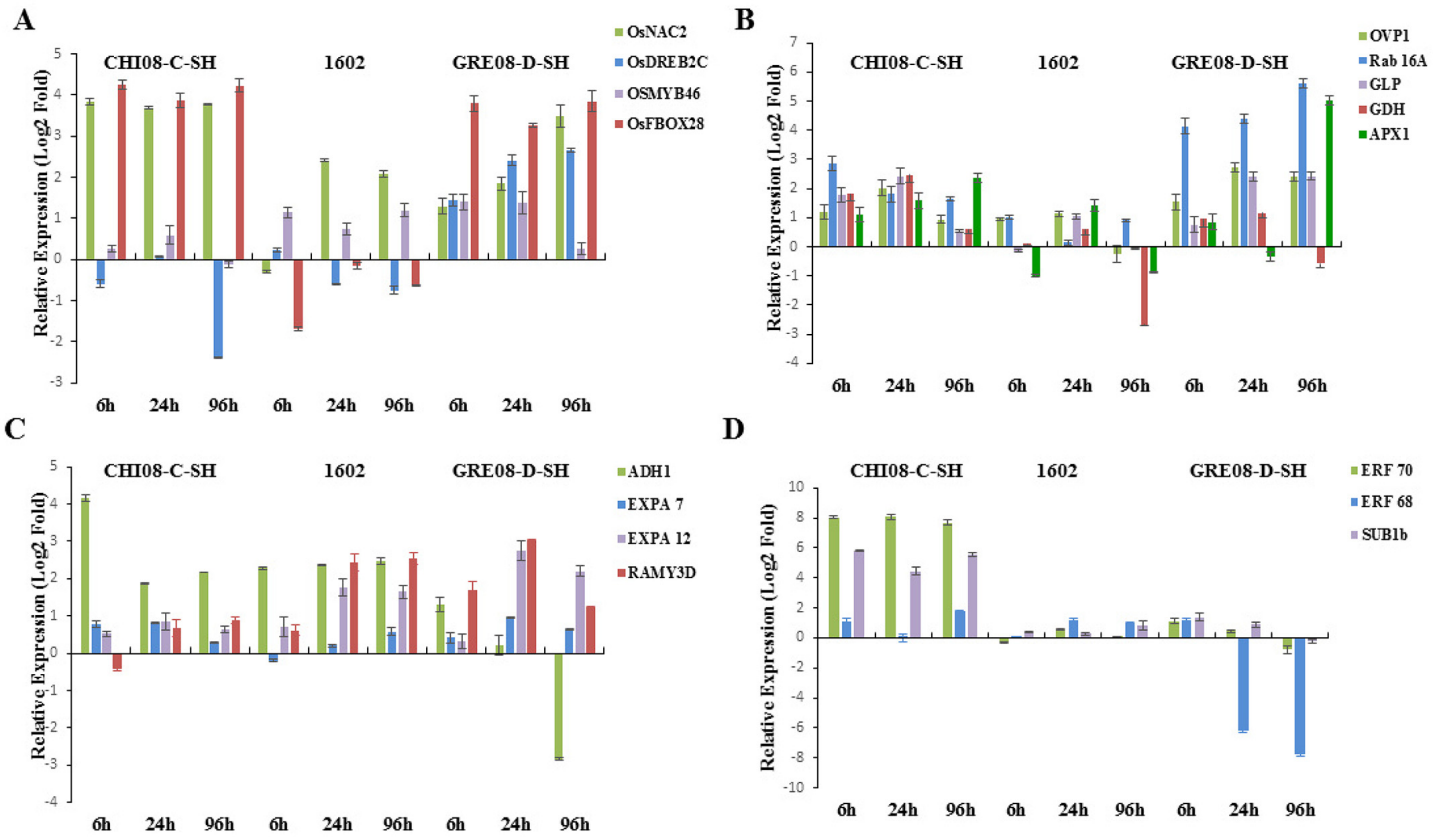
Relative expression of genes in response to combined cold stress (10°C) and depth stress (10cm) of sensitive (CHI08-C-SH) and tolerant (GRE08-D-SH and 1602) *indica* rice A. Response of transcription factors: *OsNAC2*, *OsDREB2C*, *OsMYB46* and *OsF-BOX28*. B. Response of defense- and metabolism-related genes: *OVP1*, *RAB16A*, *APX1*, *GLP*, *GDH*. C. Genes related to cell expansion and carbohydrate metabolism: *ADH1*, *EXPA7*, *EXPA12* and *RAMY3D*. D. Expression patterns of ERFs: *ERF70*, *ERF68* and *SUB1B*. The data are means of three replicates ± SE.

Rice genotypes are not known to emerge well from greater soil depth, but weedy rice ecotypes primarily belonging to the *indica* ssp. can [33–34]. Planting, or being covered, at a greater depth in the soil allows seeds to avoid temperature extremes, but there is an optimum seedling emergence depth beyond which the seedling will incur loss of vigor or perish. We studied the effect of combined stress of sowing depth (10cm) and cold, on the expression of the stress responsive genes in the rice genotypes. The cold-tolerant *japonica* cultivar Spring showed an early and generally higher transcript accumulation of all TFs (*OsDREB2C*, Os*MYB46*, *OsNAC2* and *OsF-BOX28*) than the sensitive weedy *japonica* (Fig 3A). Similarly, all TFs were highly induced in the tolerant USA weedy *indica* GRE08-D-SH whereas only *OsNAC2* and *OsF-BOX28* were highly upregulated in sensitive *indica* CHI08-C-SH (Fig 4A). Only *OsMYB46* appeared to play a role in cold + sowing depth response of the tolerant Brazilian weedy *indica* 1602.

The severity of stress was also marked by increased transcript accumulation of ROS scavenging genes *APX1* and *GLPS*. In addition, both the *indica* and *japonica* weedy rice showed enhanced transcript levels of *RAB16A* and *GDH* in contrast to *H* ^*+*^ *PYROPHOSPHATASE (OVP1)*, which was not detectable in the *japonica* ssp. (Figs 3B and 4B). These results suggest that the stress-tolerant *indica* and *japonica* genotypes activated a tolerance mechanism operating via the CBF-dependent and-independent pathways. Moreover, this indicates that the combined cold and depth stress triggered the simultaneous activation of a network of multiple physiological stresses, which eventually overcame the cold tolerance mechanism of these genotypes.

**Fig 5:**
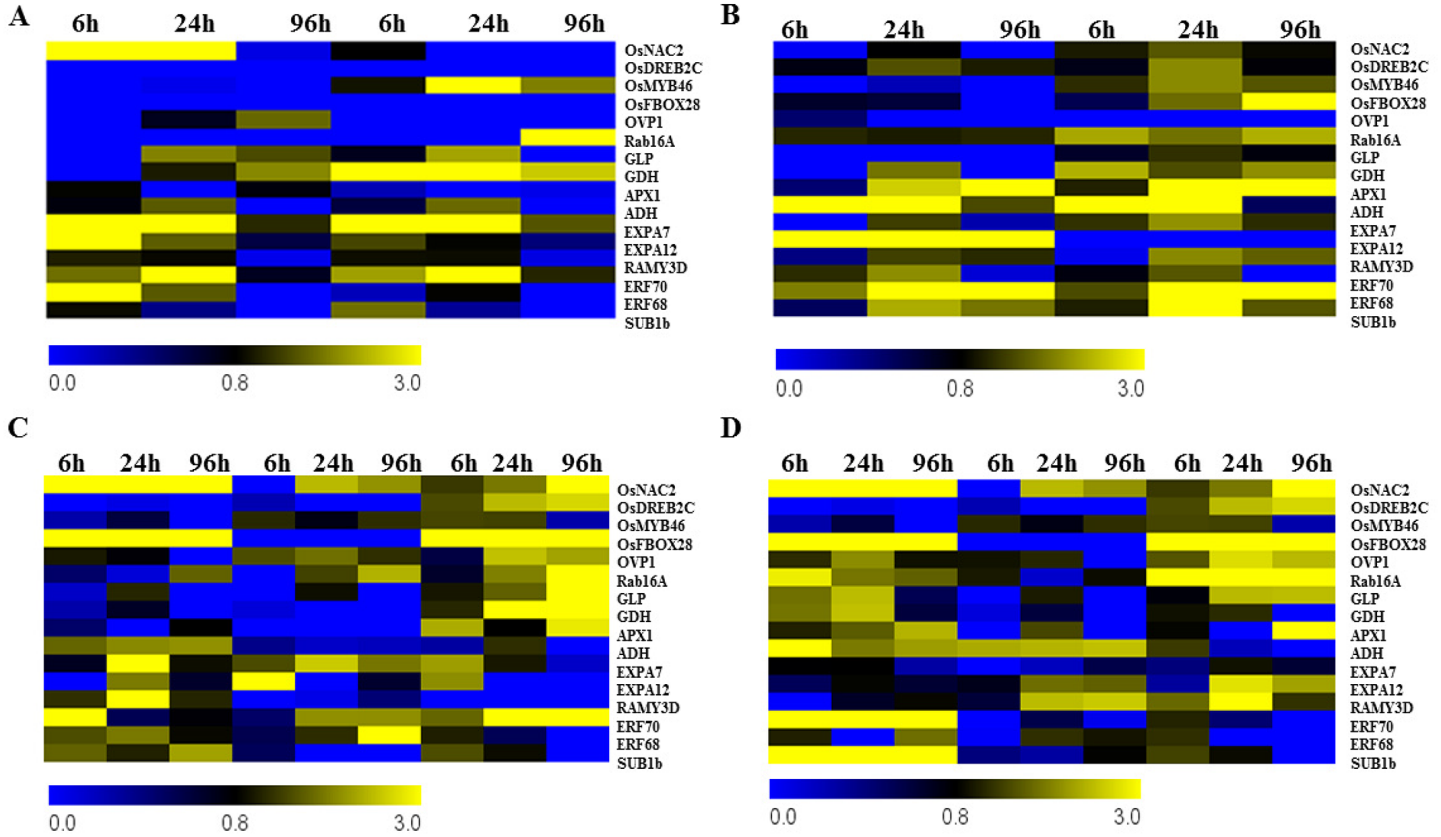
Heat Map showing the differential expression of genes in response to cold stress and combined cold + seeding depth stress in rice. Expression profile of genes in response to cold stress in: A. susceptible (PRA08-D-BH) and tolerant (Spring) *japonica* rice and B. sensitive (CHI08-C-SH) and tolerant (1602 and GRE08-D-SH) *indica* rice. Expression profile of genes in response to combinatorial stress in: C. sensitive (PRA08-D-BH) and tolerant (Spring) *japonica* rice and D. sensitive (CHI08-C-SH) and tolerant (1602 and GRE08-D-SH) *indica* rice.

**Fig 6:**
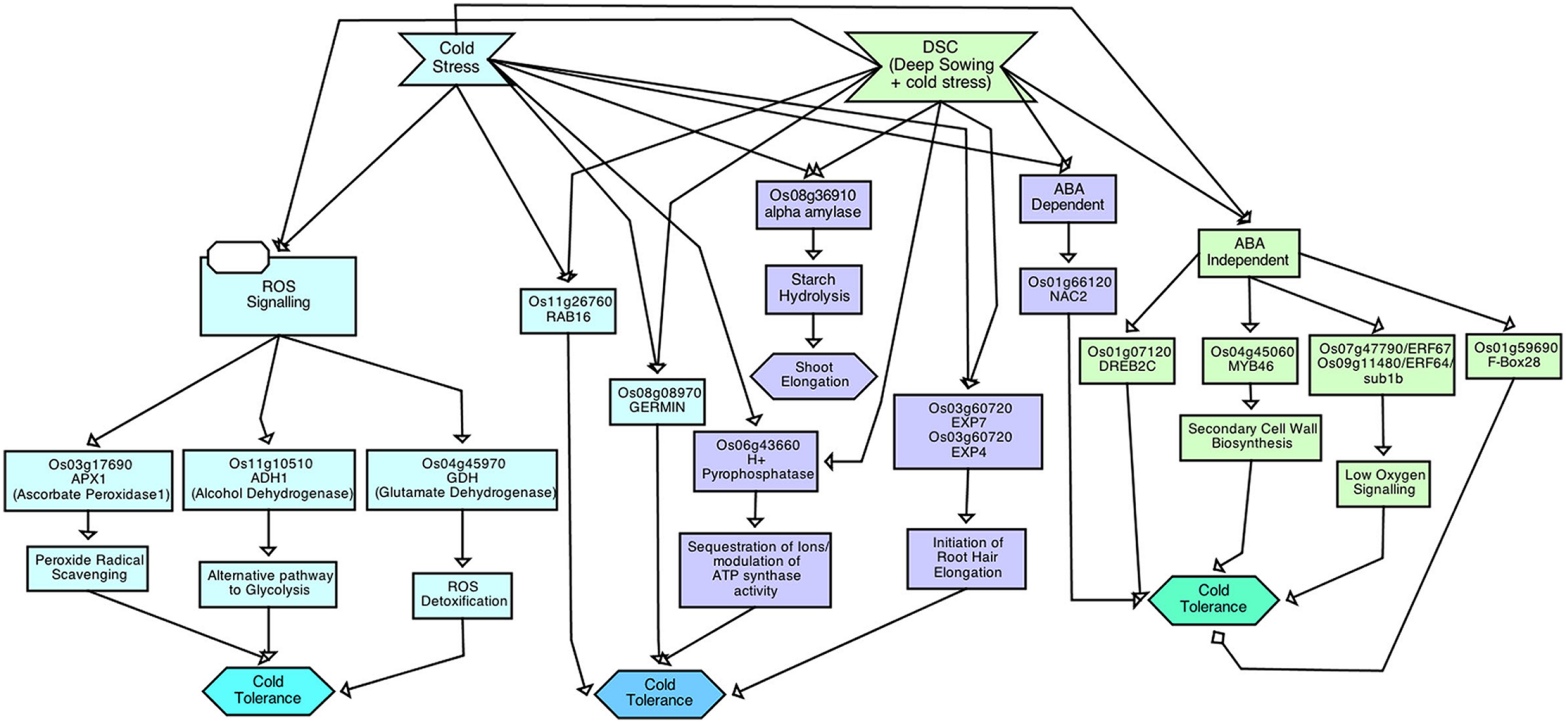
Overview of the regulatory pathway of cold stress and combination of cold and sowing depth stress response in weedy rice (*Oryza sativa* L.). The data represent the core figure drawn according to Systems Biology Graphical Notation (SBGN) Activity Flow Specifications (Level 1 Version 1) using Beacon Software76. The relationships, or arcs, express the nature of influence (positive, negative or unknown) between the glyphs and summarize this paper’s findings along with support from literature described in the text.

The tolerant *japonica* cultivar Spring showed higher accumulation of *ADH* and *alpha amylase* at 6h and 24h of stress treatment; the tolerant *indica* ssp., showed a different expression pattern (Figs 3C and 4C). *EXPA7* was generally induced equally in both ssp. while *EXPA12* was detectable in all *indica* genotypes and in the sensitive *japonica* genotype. *ERF70* and *ERF68* were highly induced in the *japonica* ssp. but only *ERF70* was detectable in the *indica* ssp. (Figs 3D and 4D). *SUB1B* was induced early at 6h in both ssp., peaked at 24h, and then declined at 96h. This suggests that the tolerant *japonica* genotypes are more vulnerable to sowing depth + cold stress than *indica*, indicating the presence of a stronger, innate tolerance mechanism in the *indica* genotypes. However, upon exposure to cold + sowing depth stress both the *indica* and *japonica* accessions showed an elevated level of all these genes suggesting that the combined stress elicited a complex network of multiple stress-response pathways. These defense/adaptive processes, however, were not sufficient to overcome the impact of the combined cold and depth stress. The overall stress responsive gene expression and pathway in weedy rice is summarized in Figs 5A–5D and 6.

## Conclusion

This research showed that there are cold-tolerant and depth-tolerant *indica* weedy rice that could be a more suitable genetic resource for the development of weed-competitive *indica* cultivars adapted to sub-tropical and temperate environments. No rice genotype tested (weedy or cultivated) can withstand cold stress if placed deep in the soil profile. The cold stress responsive gene expression of different rice and weedy rice genotypes has been summarized and represented as heat map (Fig 5A–5D). Our research also revealed that cold stress signaling in *indica* spp. is more complex than that of *japonica* as it operates via both the CBF-dependent and CBF-independent pathways implicated through increased transcript accumulation of *OsNAC2*, *OsMYB46* and *OsF-BOX28*. The *indica weedy* rice appears to have a different cold tolerance mechanism than *japonica* rice. Furthermore, cold tolerance in the USA *indica* weedy rice is by a different mechanism from the Brazilian weedy rice. These warrant further investigation. Thus, our research provided a background to identify genes responsible for cold stress tolerance in weeds. Cold-tolerant weedy rice can be tapped for rice improvement, for example by marker-assisted breeding methods. More cold-tolerant weedy rice can be discovered with a wide-scale screening of weedy rice germplasm, using molecular markers derived from this study, for developing weed-competitive rice cultivars.

## Supporting Information

S1 Table Genes used in the gene expression analysis by qRT-PCR from *indica* and *japonica* rice (*Oryza sativa* L.) genotypes exposed to cold and depth stress. (DOCX)

S1 Fig Phenol test to classify weedy rice accessions and cultivated rice as *indica* or *japonica* subspecies. The *indica* genotype has positive reaction to phenol, indicated by color change of the grain from light to dark [31]. The grain on the left-hand corner is the nontreated control. BH = black hull; SH = straw hull. Rice cultivars are 3011, Oro, Hayayuki, and Spring.
(TIFF)

S2 Fig Phenotypic change in weedy and cultivated rice seedlings in response to sowing depth stress. Seeds were germinated in the dark at 25°C for 14d. Response to depth stress was indicated by reduction in shoot growth relative to those planted at 1.5-cm depth.
(TIF)

S3 Fig Phenotypic change in weedy and cultivated rice seedlings in response to cold + sowing depth stress. Seeds were germinated in the dark at 18°C/13°C, 16/8h temperature cycle for 14d. Response to cold + depth stress was indicated by reduction in shoot growth relative to those planted at 1.5-cm depth.
(TIFF)
