# Supplementary material for: Analysis of Stress-Responsive Gene Expression in Cultivated and Weedy Rice Differing in Cold Stress Tolerance
Source: PLoS One. 2015 Jul 31;10(7):e0132100. doi: 10.1371/journal.pone.0132100 (PMC4521806; doi:10.1371/journal.pone.0132100)
Supplement: S1 Table — (DOCX) [file pone.0132100.s001.docx]

**S1 Table.** **Genes used in the gene expression analysis by qRT-PCR from *indica* and *japonica* rice (*Oryza sativa* L.) genotypes exposed to cold and depth stress**.

| **Accession** | **Primer Sequence** | **Name** | **Reference** |
| --- | --- | --- | --- |
| **AK068392** | GCAAGCCATTCTAGACGACC | NAM-F | Rabbani et al.(2003) |
|  | GCTCGCCTGAGTCAAAGTTC | NAM-R |  |
| **AK108621** | GCTCCTCGTCGACTACATCC | Myb 7-F | Rabbani et al. (2003) |
|  | CTGGACGATGGACTTCTGCT | Myb 7-R |  |
| **AK071366** | GAAGAAGGGCTTCATGGACA | Rab 16-F | Rabbani et al. (2003) |
|  | CACCATCACTCGCATTTCAC | Rab 16-R |  |
| **AK072651** | CCTGCAACATGAAGCTGAAA | F-box-F | Rabbani et al.(2003) |
|  | TCAGTTTCCTTCCGACTGCT | F-box-R |  |
| **Os03g60720** | GAGATCAAGTGCGTGAACCA | Expansin 7-F | Lasanthu-Kudahettige et al. (2007) |
|  | ACCTGAACCCGTTTATCGTG | Expansin 7-R |  |
| **Os03g44290** | AGGGATGTGGTTCGTGCTAC | Sub 1b-F | Lasanthu-Kudahettige et al. (2007) |
|  | GTCCATTCACACTCCACACG | Sub 1b-R |  |
| **Os11g10510** | AGTGTGGGAGAGGGTGTGAC | Alcohol dehydrogenase 2-F | Fukuda et al. (2005) |
|  | GTGGATGTGCCAACAAAGTG | Alcohol dehydrogenase 2-R |  |
| **Os08g36910** | AAGGTCATGGTGAAGATCGG | Alpha-amylase-F | Lasanthu-Kudahettige et al. (2007) |
|  | CCTTCTCCCAGACGCTGTAG | Alpha-amylase-R |  |
| **Os07g47790** | AGTTCATGGACTACGACGCC | Expansin 12-F | Lasanthu-Kudahettige et al. (2007) |
|  | ATCAAAGCTCCAGAGCTCCA | Expansin 12-R |  |
| **Os01g21120** | ACTACATGAGCTTCCTCGGC | ERF 68-F | Nakano et al. (2006) |
|  | GACGGCAGCTCGTAGTCTTC | ERF 68-R |  |
| **Os03g17690** | AGGTGCCACAAGGAAAGATCTGGT | APX2-F | Shigeoka et al. (2002) |
|  | TCAGCAGGGCTTTGTCACTAGGAA | APX2-R |  |
| **Os09g11480** | GGACGCCACAACGAAGATGAAGAA | ERF 70-F | Nakano et al. (2006). |
|  | TGCACCAGAAGGGAACATGGAAAC | ERF 70-R |  |
| **AF300971** | TAAGTGGGTGGCTGAGATCC | Dreb2A-F | Dubouzet et al. (2003) |
|  | ATGAAGGTGCTGATGTGCAG | Dreb2A-R |  |
| **BP432999** | TTTTCATGATGCGGAAGTCA | Glutamate dehydrogenase-F | Rabbaniet al. (2003) |
|  | TTTTCCCTGTTGAGCACTCC | Glutamate dehydrogenase-R |  |
| **Os08g08970** | CATCTCACTAGCTCGCATCG | Germin-F | Dubouzet et al. (2003) |
|  | TGTTCCCTCAAGCACAGTGA | Germin-R |  |
| **D45383** | TTGAGCCTGCCCTCAAGAAG | H+ pyrophosphatase-F | Rabbani et al. (2003) |
|  | GGGAGGCCTAACCAACTGAC | H+ pyrophosphatase-R |  |
| **UBI** | CTCACCTACGTCTACAA | Ubiquitina -F | Jain et al.  (2006) |
|  | GTCAAGGTGTTCAGTTC | Ubiquitina -R |  |
