# Supplementary figures and images for: Analysis of Stress-Responsive Gene Expression in Cultivated and Weedy Rice Differing in Cold Stress Tolerance
Source: PLoS One. 2015 Jul 31;10(7):e0132100. doi: 10.1371/journal.pone.0132100 (PMC4521806; doi:10.1371/journal.pone.0132100)

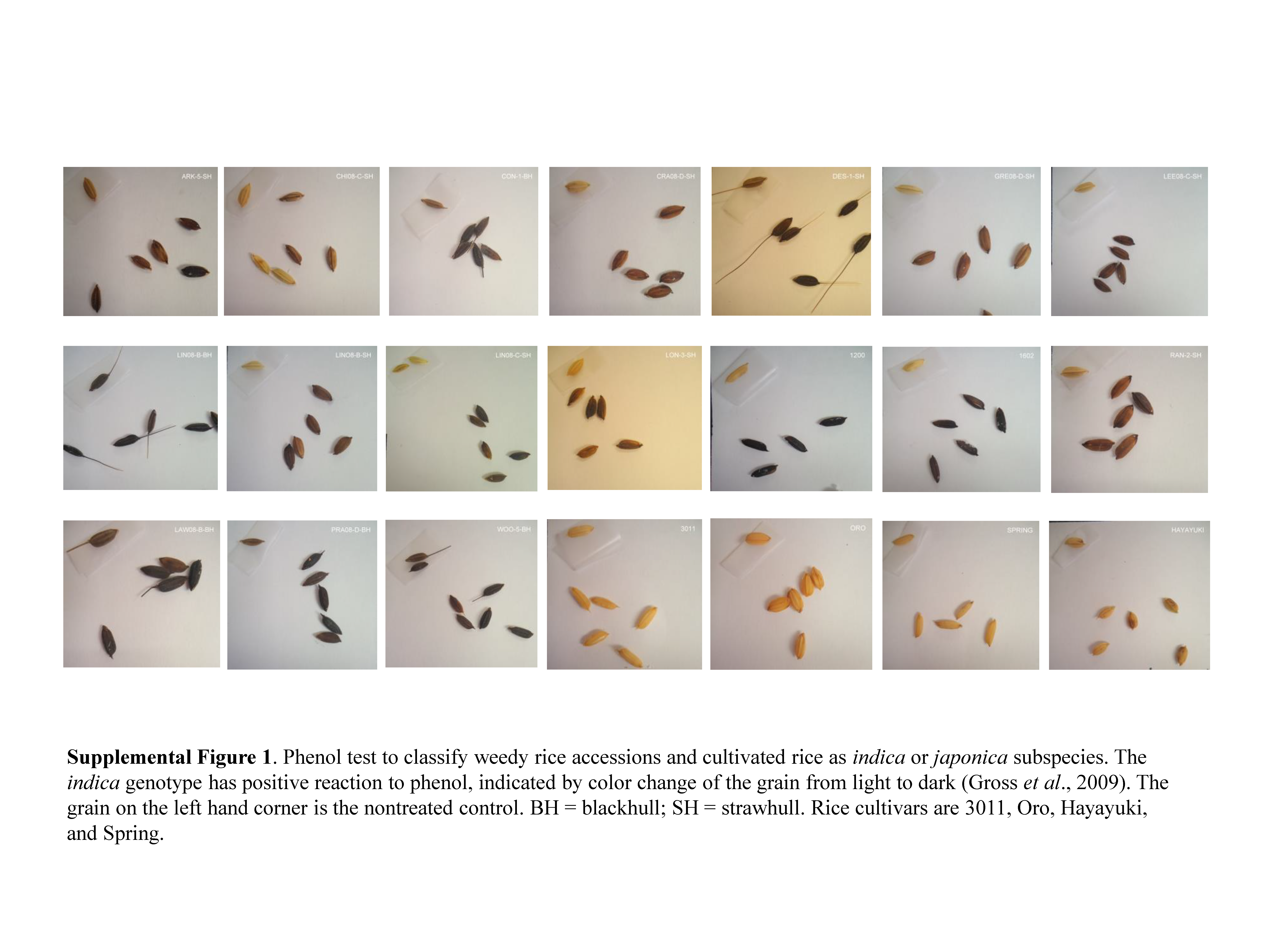

Supplement: S1 Fig — The indica genotype has positive reaction to phenol, indicated by color change of the grain from light to dark [31]. The grain on the left-hand corner is the nontreated control. BH = black hull; SH = straw hull. Rice cultivars are 3011, Oro, Hayayuki, and Spring. (TIFF) [file pone.0132100.s002.tiff]

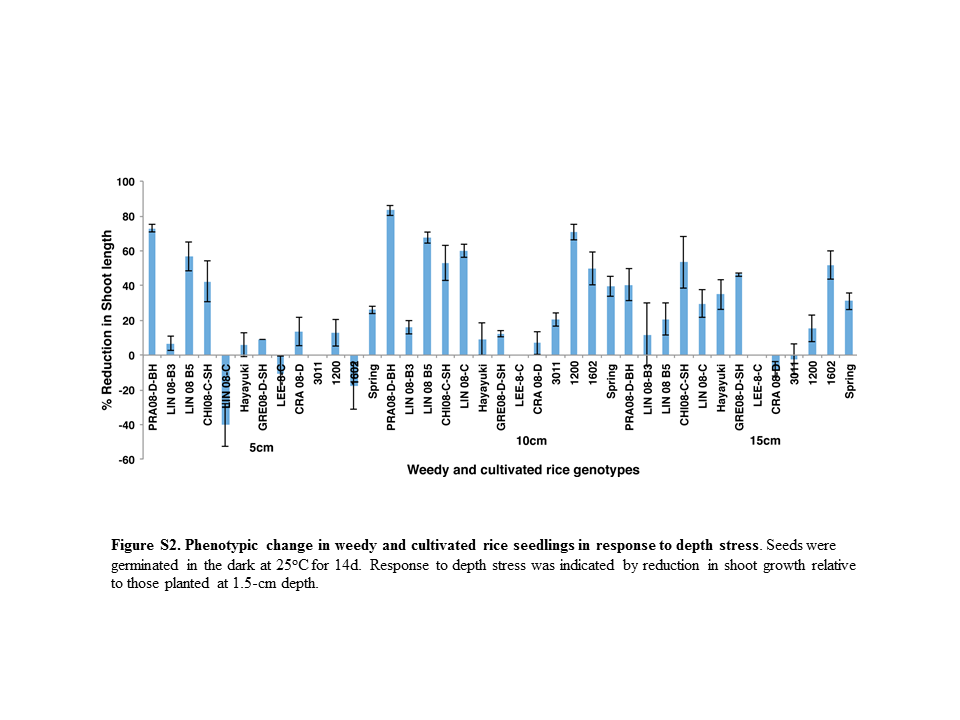

Supplement: S2 Fig — Seeds were germinated in the dark at 25°C for 14d. Response to depth stress was indicated by reduction in shoot growth relative to those planted at 1.5-cm depth. (TIF) [file pone.0132100.s003.tif]

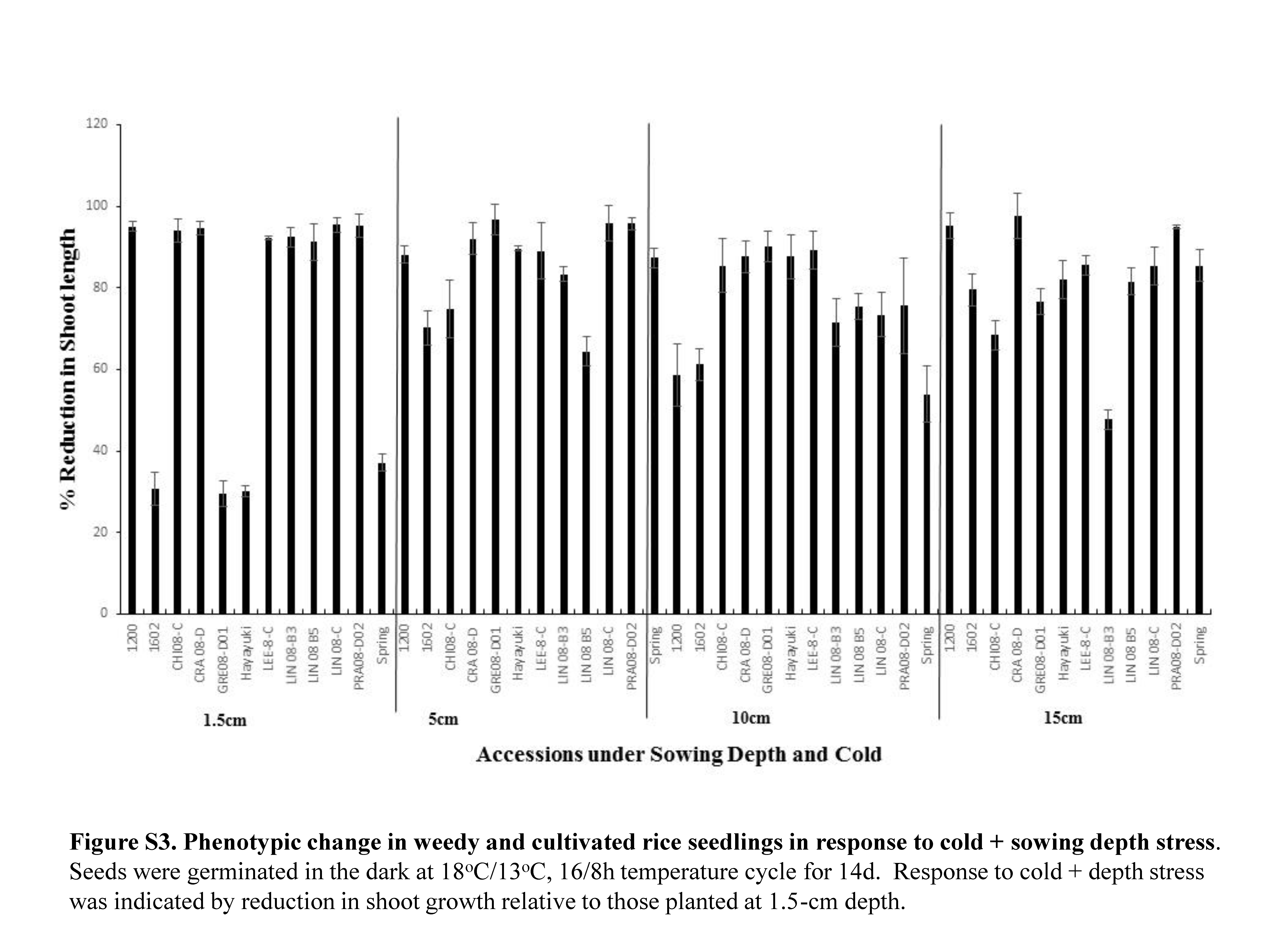

Supplement: S3 Fig — Seeds were germinated in the dark at 18°C/13°C, 16/8h temperature cycle for 14d. Response to cold + depth stress was indicated by reduction in shoot growth relative to those planted at 1.5-cm depth. (TIFF) [file pone.0132100.s004.tiff]
